# Clinical characteristics and genotype‐phenotype correlations of 130 Chinese children in a high‐homogeneity single‐center cohort with 5α‐reductase 2 deficiency

**DOI:** 10.1002/mgg3.1431

**Published:** 2020-07-26

**Authors:** Lijun Fan, Yanning Song, Michel Polak, Lele Li, Xiaoya Ren, Beibei Zhang, Di Wu, Chunxiu Gong

**Affiliations:** ^1^ Department of Endocrinology, Genetics, Metabolism Beijing Children’s Hospital Capital Medical University National Center for Children’s Health Beijing China; ^2^ Beijing Key Laboratory for Genetics of Birth Defects Beijing Children’s Hospital Capital Medical University National Center for Children’s Health Beijing China; ^3^ Service d'endocrinologie, gynécologie et diabétologiepédiatriques Hôpitaluniversitaire Necker Enfants Malades université de Paris IMAGINE institute Paris France

**Keywords:** disorders of sex development, genotype‐phenotype correlation, *SRD5A2* gene, steroid 5α‐reductase type 2 deficiency

## Abstract

**Background:**

Patients with steroid 5α‐reductase 2 deficiency (5α‐RD) caused by *SRD5A2* (OMIM #607306) variants present variable genotypes and phenotypes. The genotype‐phenotype correlations remain unclear.

**Methods:**

We investigated genotype‐phenotype correlations of *SRD5A2* variants in a large Chinese single‐center cohort. Phenotypes were categorized using the external masculinization score (EMS), urethral meatus and gonad position, and penile length‐standard deviation score.

**Results:**

Of the 130 included patients, 113 had hypospadias, and 17 had a normal urethral meatus position. Testosterone/dihydrotestosterone (T/DHT) values were not significantly associated with phenotypic severity (*p* = 0.539–0.989). Of the 31 *SRD5A2* variants, including 10 novel variants, p.R227Q was the most prevalent (39.62%), followed by p.Q6* (16.92%), p.R246Q (13.46%), and p.G203S (10.38%). Compared to biallelic missense mutations, biallelic nonsense mutations were associated with a lower EMS and urethral meatus score (*p* = 0.009 and *p* = 0.024, respectively). Patients homozygous for p.R227Q exhibited mild and variable phenotypes, while those homozygous for p.Q6*, p.R246Q, or p.G203S showed consistently severe phenotypes. The phenotypes were variable and milder in patients with compound heterozygosity for p.R227Q and these mutations.

**Conclusion:**

T/DHT does not predict phenotype severity. The most prevalent *SRD5A2* variant in Han Chinese is p.R227Q, which is associated with milder phenotypes and greater phenotypic variability. *SRD5A2* variants may significantly influence phenotypic variation.

## Graphical abstract

Patients homozygous for p.R227Q exhibited mild and variable phenotypes while those with homozygous p.Q6*, p.R246Q, and p.G203S showed consistently severe phenotypes. When those mutations were compound heterozygotes for p.R227Q, the genotypes were variable and milder.

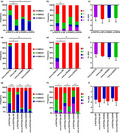

## 1 INTRODUCTION

Steroid 5α‐reductase 2 deficiency (5α‐RD; OMIM #264600) is an autosomal recessive 46,XY disorder of sex differentiation (DSD) caused by variants of the *SRD5A2* gene (OMIM #607306) that manifests variable degrees of undervirilization. *SRD5A2*, the gene on chromosome 2p23 that encodes 5α‐reductase 2, is expressed in the fetal genital skin, male accessory sex glands, and prostate (Thigpen et al., 1993). The enzyme 5α‐reductase converts testosterone (T) to the more active androgen dihydrotestosterone (DHT). While T plays a crucial role in transforming the Wolffian ducts into the epididymis, vasa deferens, seminal vesicles, and ejaculatory ducts, DHT is essential for the normal in utero development of the male external genitalia, prostate, and urethra (Cheon, 2011). Therefore, 5α‐RD manifests as failed or incomplete masculinization of external genitalia with normal Wolffian duct structures.

The phenotypic variability of 5α‐RD ranges from perineal hypospadias to isolated micropenis (Gad, Nasr, Mazen, Salah, & el‐Ridi, 1997; Maimoun et al., 2011), and this variability has been linked to differences in *SRD5A2* gene mutations (Abaci et al., 2019). Nonetheless, the correlations between phenotype, T/DHT and genotype remain unclear for the following reasons: it is difficult to collect enough patients in single‐center studies, and multicenter studies are limited by heterogeneity in phenotypes, which are difficult to quantitate, and hormone detection methods. In 2000, Ahmed et al. (Ahmed, Khwaja, & Hughes, 2000) proposed the external masculinization score (EMS), which provides an objective method to evaluate the appearance of external genitalia. In this large, highly homogenous Chinese single‐center cohort with documented *SRD5A2* defects, we used EMS as the main phenotypic descriptor and profiled genotype‐phenotype correlations, aiming to provide evidence for accurate diagnosis and individualized management.

## 2 PATIENTS AND METHODS

### 2.1 Editorial policies and ethical considerations

The research protocol was approved by the ethics committee of Beijing Children's Hospital, Capital Medical University (ID: 2012–28), and written informed consent was received from all patients or legal guardians.

### 2.2 Patients

We studied consecutive Chinese children belonging to the Han ethnic group who received a definitive diagnosis of 5α‐RD at Beijing Children's Hospital between December 2007 and May 2019. Inclusion criteria were normal morphology except for external genitalia; the presence of testicles and absence of Müllerian structures by ultrasonography; a male hormone profile; 46,XY karyotype; homozygous or compound heterozygous *SRD5A2* variants classified to be pathogenic or likely pathogenic according to the American College of Medical Genetics and Genomics and the Association for Molecular Pathology (ACMG/AMP) guidelines; and negative genetic tests for 46,XY DSDs other than 5α‐RD, such as defects in *SRY*, *AR*, *NR5A1*, and other genes.

### 2.3 Phenotypes and genotype‐phenotype correlations

**Figure 1:**
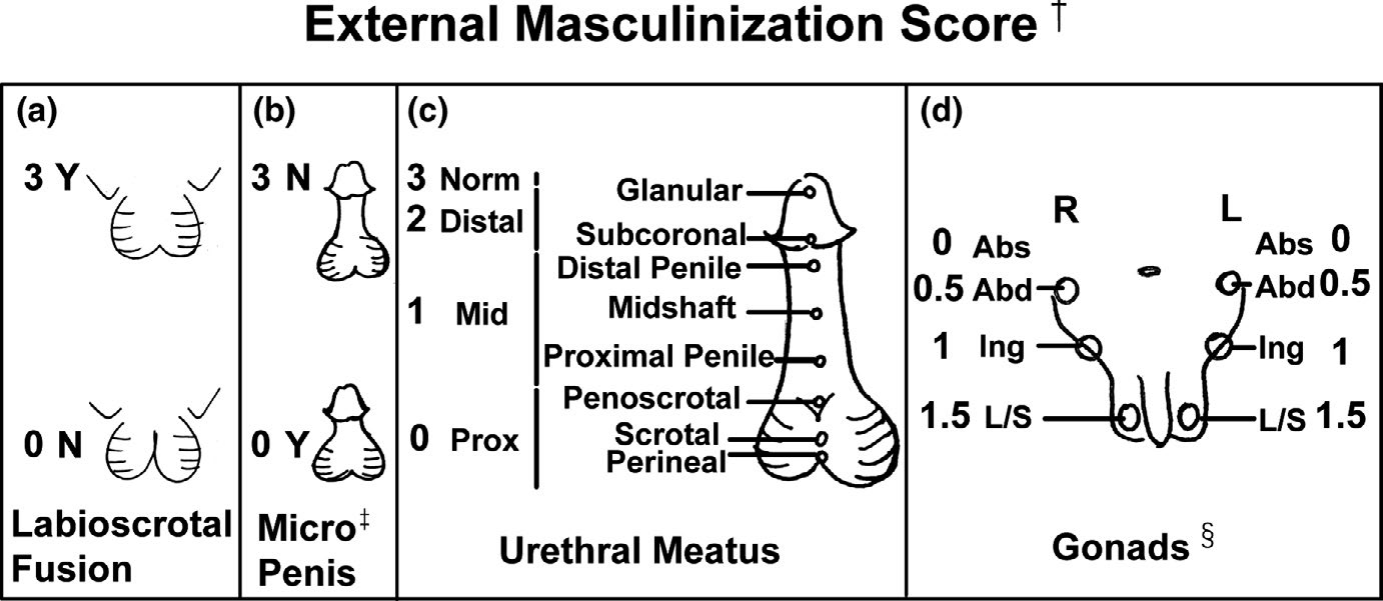
The external masculinization score (EMS). External genitalia were scored separately for scrotal fusion (a), penis size (b), location of the urethral meatus (c), and site of gonads (d). The EMS is the sum of the four subscores and can range from 0 to 12. ^†^According to Ahmed SF et al. (Ahmed & Rodie, 2010), ^‡^micropenis is defined as a stretched penile length <2.5 SDs below the mean for normal same‐age individuals. ^§^In most patients with cryptorchidism, the testes are located in the inguinal region, and we consequently defined a severe phenotype as ≤2 points, namely, bilateral inguinal cryptorchidism or other more serious phenotypes; a moderate phenotype as 2.5, namely, unilateral inguinal cryptorchidism; and bilateral descended testes as 3.EMS > 6, 3 < EMS ≤ 6, and EMS ≤ 3 defined mild, moderate, and severe phenotypes, respectively. Abd, Abdominal; Abs, Absence; Distal, Distal hypospadias; Ing, Inguinal; L/S, Labia/Scrotum; Mid, Midshaft hypospadias; N, No; Norm, Normal urethral meatus; Prox, Proximal hypospadias; Y, Yes

We compared the genotypes in the groups with and without hypospadias and the phenotypes among groups defined by variant sites and genotypes. Phenotypes were classified based on the EMS (Ahmed et al., 2000; Ahmed & Rodie, 2010), subscores for urethral meatus and gonad location (Figure 1) and on the penile length‐standard deviation score (PL‐SDS) at the first visit. All external genital examinations were performed by the same endocrinologist (Chunxiu Gong). Stretched PL was measured as previously described (Lee et al., 2012) and compared to reference values obtained in China (Fu & Li, 2010) (Table S1).

### 2.4 Endocrine investigations

Basal luteinizing hormone (LH) and follicle‐stimulating hormone (FSH) levels were determined. Mini‐puberty was defined as an active hypothalamic‐pituitary‐gonadal axis with elevated T levels in infancy (Kurtoglu & Bastug, 2014). Basal T and DHT levels were measured in patients in mini‐puberty or puberty, whereas prepubertal patients underwent T and DHT testing 1 day after the daily administration of 1500 IU of human chorionic gonadotropin (hCG) on four consecutive days. All measurements were performed before surgical treatment. LH, FSH, and T values were measured by chemiluminescent immunoassay (Immulite 2000, Siemens Healthcare, Sudbury, UK), and DHT was measured by liquid chromatography‐mass spectrometry (1200 Series HPLC system, Agilent, Santa Clara, CA; API5000 tandem mass spectrometer, AB Sciex Pte, Framingham, MA). The interassay and intraassay coefficients of variation were less than 10%.

### 2.5 Genetic tests

Karyotyping was performed for all patients. Genetic testing consisted of targeted Sanger sequencing of four DSD‐related genes (*SRD5A2*, *AR*, *NR5A1*, and *SRY*) and next‐generation sequencing (NGS) of a panel of 127 DSD‐related genes or whole‐exome sequencing (WES), depending on the clinical features and financial resources of the patients. For Sanger sequencing, *SRD5A2* exons 1‐5 and exon‐intron boundaries were amplified by PCR using previously described primers (Baldinotti et al., 2008). NGS was performed by MyGenostics (Beijing, China) or the Beijing Kangso Medical Laboratory. When screening and analysis identified candidate variants, Sanger sequencing and parental validation were performed. All data interpretation was based on the GRCh37/hg19 human genome assembly, and the reference sequence for *SRD5A2* was NM_000348.3.

### 2.6 Predicted effects of *SRD5A2* variants

The potential pathogenicity of variants was classified according to ACMG/AMP guidelines (Richards et al., 2015). In silico analysis was performed to assess the impact of the novel mutations on protein structure using the web software Polyphen‐2 (http://genetics.bwh.harvard.edu/pph2/).

### 2.7 Statistical analysis

Data are presented as the mean ± SD or median [interquartile range, IQR] as appropriate. Statistical analyses were performed using SPSS version 22.0 (IBM, Armonk, NY). Graphs were plotted using GraphPad Prism version 7.0 (San Diego, CA) and Adobe Illustrator CS6 version 16.0 (San Jose, CA). Normally distributed data were compared between groups using the *t*‐test or one‐way ANOVA followed by Bonferroni's correction, and nonnormally distributed data were compared using the Mann–Whitney U test or Kruskal–Wallis test followed by the Dunn–Bonferroni post hoc test. Correlations between variables were evaluated by computing Spearman's correlation coefficients. The chi‐square test was chosen to assess differences in proportions. Two‐tailed *p* values <0.05 were considered to indicate statistical significance.

## 3 RESULTS

We studied 130 patients, including six pairs of siblings. The median patient age was 1.4 years [0.7–3.5] at the first visit and 1.9 years [0.8–4.4] at diagnosis.

### 3.1 Phenotypes

At the first visit, 93 patients with predominately male external genitalia were assigned male, including 76 with hypospadias and micropenis and 17 with micropenis; 37 patients with female external genitalia were assigned female, including 30 with clitoromegaly and seven with complete female genitalia and masses in the groin or labia. After diagnosis, 35 patients had their assigned sex changed from female to male; two siblings were reared as females and underwent gonadectomy. The final sex assignment was male in 128 cases and female in two cases.

**Figure 2:**
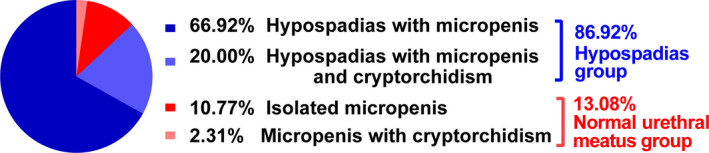
Clinical characteristics of 130 Chinese children with *SRD5A2* mutations

Most patients (*n* = 113) had hypospadias, either with micropenis only (*n* = 87) or with both micropenis and cryptorchidism (*n* = 26). Of the 17 patients with a normal urethral meatus position, 14 had micropenis, and three had both micropenis and cryptorchidism (Figure 2). Table 1 shows the phenotypic scores of all subjects.

**Table 1:** Phenotypes in 130 Chinese children with *SRD5A2* gene variants

Phenotype	Classification	Frequency
EMS	0 < EMS ≤ 3	41.54% (54/130)
	3 < EMS ≤ 6	16.15% (21/130)
	6 < EMS ≤ 12	42.31% (55/130)
Median [IQR]	6 [3–7]	
Urethral meatus score	0	51.54% (67/130)
	1	27.69% (36/130)
	2	7.69% (10/130)
	3	13.08% (17/130)
Median [IQR]	1 [0‐1]	
Gonad position score	≤2	10.77% (14/130)
	2.5	12.31% (16/130)
	3	76.92% (100/130)
Median [IQR]	3 [2.5–3]	
PL‐SDS (male sex)^a^		
Mean ± SD	−4.74 ± 1.39	
PL‐SDS (female sex)^a^		
Mean ± SD	−5.88 ± 1.77	
PL‐SDS (all patients)		
Mean ± SD	−5.08 ± 1.59	

Abbreviations: EMS, external masculinization score; IQR, interquartile range; PL‐SDS, penile length‐standard deviation score.

^a^ Sex assigned at the first visit.

### 3.2 Endocrine investigations

The T/DHT ratio was determined in the 90 patients seen after the DHT assay became available at our center in 2014. Of the 77 prepubertal patients who underwent the hCG stimulation test, 76 and 71 had T/DHT ratios above 10 and 15, respectively. With either cutoff, the T/DHT ratio indicated 5α‐RD in five of the eight patients in mini‐puberty. Of the five patients undergoing puberty, five and four had T/DHT ratios above 10 and 15, respectively (Table 2).

**Table 2:** Hormone levels in patients with *SRD5A2* gene mutations

Hormones	Mini‐puberty, *n* = 11		Prepuberty, *n* = 109		Puberty, *n* = 10	
	*N* ^a^	Range	*N* ^a^	Range	*N* ^a^	Range
LH (IU/L)	11	3.79 ± 1.59	98	0.15 [0.10‐0.34]	8	4.59 ± 3.41
FSH (IU/L)	11	3.04 ± 1.51	98	0.84 [0.56‐1.18]	8	3.20 [2.85‐6.68]
T (ng/dl)	10	224.07 ± 87.65	98 ^b^	359.50 [215.75‐523.50]	8	320.60 ± 162.62
DHT (pg/ml)	8	158.18 ± 82.67	78 ^b^	107.05 [66.48‐190.00]	5	162.80 ± 129.86
T/DHT	8	19.47 ± 10.87	77 ^b^	29.23 [19.06‐37.66]	5	31.88 ± 16.23
T/DHT > 10	5/8		76/77		5/5	
T/DHT > 15	5/8		71/77		4/5	

Abbreviations: DHT, dihydrotestosterone; FSH, follicle‐stimulating hormone; hCG, human chorionic gonadotropin; LH, luteinizing hormone; T, testosterone.

^a^ Number of patients who underwent the relevant testing.

^b^ T and DHT were measured after hCG stimulation in prepubertal patients (the remaining hormone levels are basal values). Data are presented as the mean ± SD or median [interquartile range, IQR], as appropriate. Reference intervals: LH: 0–4.1 IU/L (1–17 months), 0–3.8 IU/L (1.5–8 years), 0–7.6 IU/L (9–14 years), and 0.8–7.6 IU/L (15–44 years); FSH: 0–5.5 IU/L (1 months to 2 years), 0–1.9 IU/L (3–8 years), 0–11.1 IU/L (9–14 years), and 0.7–11.1 IU/L (15–44 years); T: 0–106.9 ng/dl (0–11 months), 0–21.2 ng/dl (1–4 years), 0–38.7 ng/dl (5–9 years), 21.1–54.6 ng/dl (10–11 years), and 114.6–291.4 ng/dl (12–13 years); DHT: 106–719 pg/ml.

Of the 17 patients without hypospadias, 10 underwent an hCG stimulation test, which consistently produced a T/DHT ratio above 15 (mean, 37.59 ± 18.24; *p* = 0.903 compared to the group with hypospadias). No significant correlations were found between stimulated T/DHT values and phenotypic features (EMS, *p* = 0.829; urethral meatus score, *p* = 0.722; gonad location score, *p* = 0.989; and PL‐SDS, *p* = 0.539).

### 3.3 *SRD5A2* mutations

**Figure 3:**
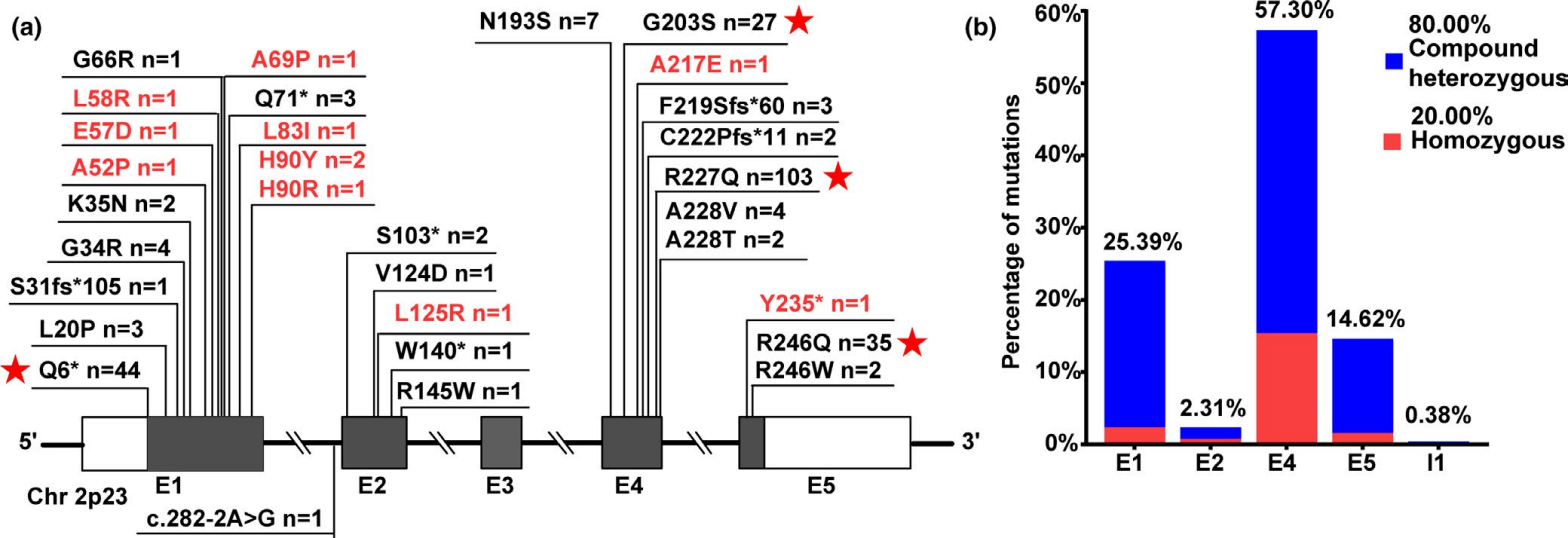
The *SRD5A2* mutational spectrum in 130 Chinese children with 5α‐RD. (a) Identified *SRD5A2* variants and frequencies. Red font indicates unreported variants, black font indicates reported variants, and red stars indicate common variants. (b) Distribution of variants throughout the *SRD5A2* gene. E, Exon; I, Intron

Of the 130 patients, 67 underwent targeted Sanger sequencing of the *SRD5A2* gene, and 63 underwent NGS (30 by DSD‐related gene panel assays and 33 by WES). We identified 31 *SRD5A2* variants, of which 10 were previously unreported. Homozygous and compound heterozygous *SRD5A2* mutations were present in 26 (20.00%) and 104 (80.00%) patients, respectively. Twenty‐two variants were located in exons 1 and 4 of *SRD5A2*, accounting for 215 alleles (82.69%). Missense variants (*n* = 202 alleles, 77.69%) and nonsense variants (*n* = 51 alleles, 19.62%) were responsible for the majority of the allelic alterations, whereas frameshift variants (*n* = 6 alleles, 2.31%) and splice site variants (*n* = 1 allele, 0.38%) were rare. The most prevalent variant was p.R227Q (*n* = 103 alleles, 39.62%), followed by p.Q6* (*n* = 44 alleles, 16.92%), p.R246Q (*n* = 35 alleles, 13.46%), and p.G203S (*n* = 27 alleles, 10.38%) (Figure 3, Table S2). According to ACMG/AMP guidelines, the novel missense variants and nonsense variant were classified as likely pathogenic and pathogenic, respectively. The novel missense variants were predicted to be probably damaging by Polyphen‐2 software (Table S3).

### 3.4 Genotype‐phenotype correlations

We analyzed phenotypic differences based on the different types of variants in the coding sequence and excluded patients with splice variants of the *SRD5A2* gene from further studies. There were no significant phenotypic differences between patients with homozygous mutations and those with compound heterozygous mutations (*p* = 0.203–0.878). All five patients with biallelic nonsense mutations had a severe phenotype, with EMS ≤3 and severe hypospadias; their EMS and urethral meatus scores were lower than those of patients with biallelic missense mutations (*p* = 0.009 and *p* = 0.024, respectively). Five patients had compound heterozygosity for frameshift mutations and other variants, of which three had severe phenotypes and two had moderate phenotypes (Table 3).

**Table 3:** Phenotypes of patients with frameshift, biallelic missense, or nonsense variants

Group	Genotype	*N*	EMS (median)	Urethral meatus (median)	PL‐SDS (mean)
Biallelic missense variants	p.R227Q/p.R246Q	18	7	1	−4.73
	p.R227Q/p.R227Q	15	7	1	−4.30
	p.R227Q/p.G203S	8	7.75	2	−4.36
	p.G203S/p.G203S	5	2.5	0	−5.74
	p.R227Q/p.N193S	5	7	1	−6.02
	p.R227Q/p.A228V	3	8	2	−5.12
	p.R246Q/p.R246Q	2	3	0	−5.72
	p.R227Q/p.K35N	2	2.25	0	−6.38
	p.R227Q/p.G34R	2	4	0	−4.11
	p.G203S/p.R246Q	2	5	0.5	−4.86
	p.R227Q/p.L20P	2	6.5	0.5	−6.02
	p.G66R/p.H90Y	1	3	0	−7.86
	p.R227Q/p.A52P	1	9	3	−2.76
	p.R227Q/p.E57D	1	2	0	−4.43
	p.R227Q/p.H90R	1	6	0	−4.71
	p.R227Q/p.H90Y	1	7	1	NA
	p.R227Q/p.L58R	1	7	1	−4.14
	p.R227Q/p.L83I	1	3	0	NA
	p.R227Q/p.R145W	1	9	3	−4.14
	p.R227Q/p.R246W	1	3	0	−4.14
	p.R227Q/p.V124D	1	3	0	−4.14
	p.A228V/p.R246Q	1	3	0	−5.41
	p.G203S/p.G34R	1	2.5	0	−7.29
	p.G203S/p.L125R	1	5.5	0	NA
	p.G203S/p.L20P	1	3	0	−3.06
	p.R246W/p.R246Q	1	2.5	0	−3.67
Biallelic nonsense variants	p.Q6*/p.Q6*	3	2	0	−5.27
	p.S103*/p.S103*	1	1	0	−8.14
	p.Q6*/p.W140*	1	3	0	NA
Frameshift variants	p.F219Sfs*60/p.R227Q	1	6	0	−5.29
	p.F219Sfs*60/p.R246Q	1	3	0	−5.29
	p.F219Sfs*60/p.N193S	1	3.5	1	−5.54
	p.C222Pfs*11/p.R227Q	1	3	0	−3.67
	p.C222Pfs*11/p.Q6*	1	2	0	−3.06

Abbreviations: EMS, external masculinization score; N, number of patients; NA, data not available; PL‐SDS, penile length‐standard deviation score; Urethral meatus, urethral meatus score.

**Figure 4:**
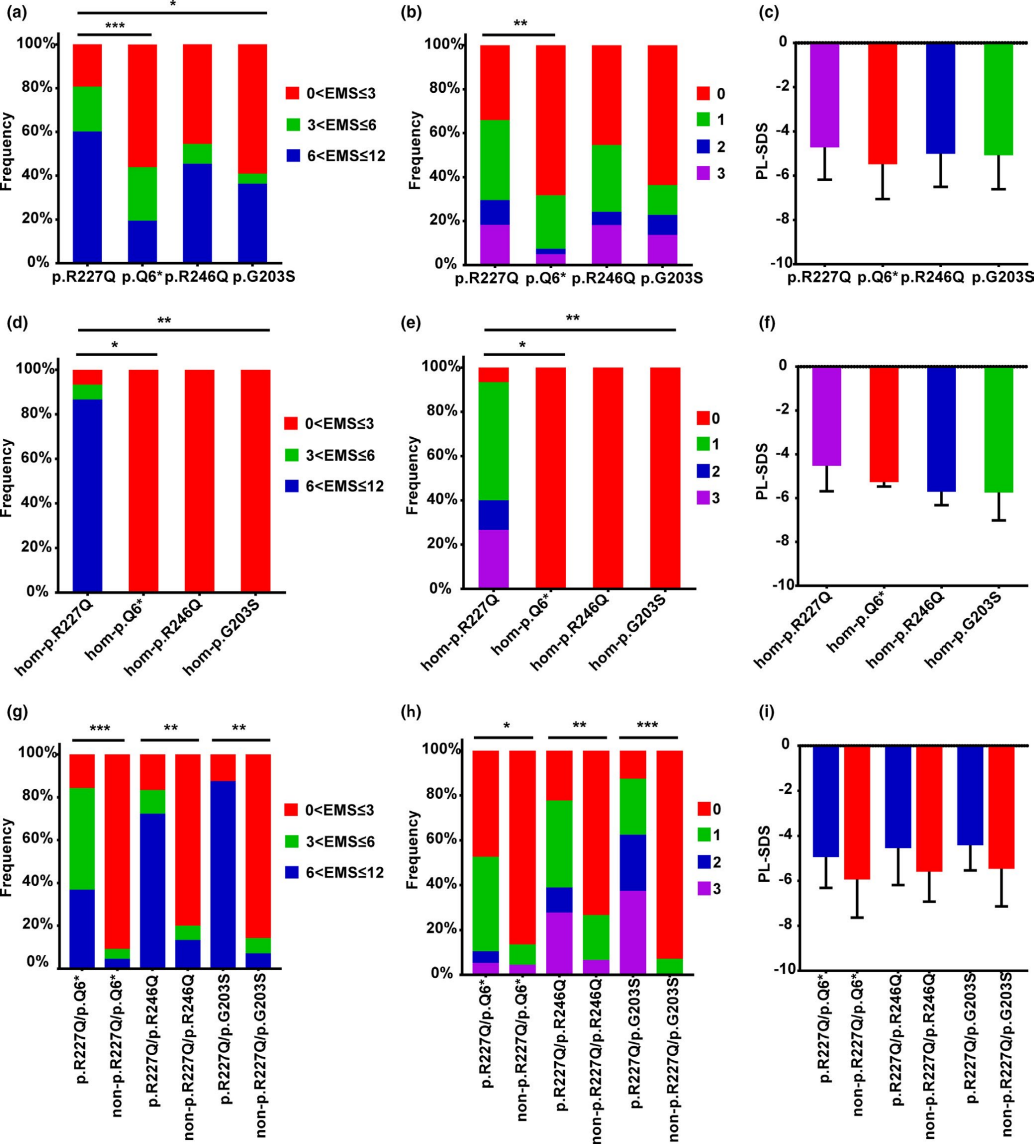
Comparison of phenotypes across *SRD5A2* genotypes. (a–c) EMS (a), urethral meatus position (b), and PL‐SDS (c) in patients with p.R227Q (*n* = 88), p.Q6* (*n* = 41), p.R246Q (*n* = 33), and p.G203S (*n* = 22). *p* values from left to right: (a) *p* < 0.001, *p* = 0.024; (b) *p* = 0.001. (d–f) EMS (d), urethral meatus position (e), and PL‐SDS (f) in patients with different homozygous *SRD5A2* mutations (p.R227Q, *n* = 15; p.Q6*, *n* = 3; p.R246Q, *n* = 2; and p.G203S, *n* = 5). *p* values from left to right: (d) *p* = 0.027, *p* = 0.003; (e) *p* = 0.045, *p* = 0.006. (g–i) EMS (g), urethral meatus position (h), and PL‐SDS (i) in patients with or without p.R227Q within the subgroup carrying other high‐frequency mutations (p.R227Q/p.Q6*, *n* = 19; non‐p.R227Q/p.Q6*, *n* = 22; p.R227Q/p.R246Q, *n* = 18; non‐p.R227Q/p.R246Q, *n* = 15; p.R227Q/p.G203S, *n* = 8; and non‐p.R227Q/p.G203S, n = 14). *p* values from left to right: (g) *p* < 0.001, *p* = 0.001, *p* = 0.001; (h) *p* = 0.011, *p* = 0.006, *p* < 0.001. Data are presented as the mean ± SD. Statistical analysis: Kruskal–Wallis test followed by the Dunn–Bonferroni test for EMS and urethral meatus position; one‐way ANOVA followed by Bonferroni's correction for PL‐SDS. EMS, external masculinization score; hom, homozygous; PL‐SDS, penile length‐standard deviation score. ***p < 0.001, **p < 0.01, *p ≤ 0.05

Figure 4a–c shows the correlations between recurrent mutations and phenotypes. The p.Q6* mutation was associated with lower EMS and urethral meatus scores than the p.R227Q mutation (*p* < 0.001 and *p* = 0.001, respectively). The EMS was lower in the patients with p.G203S than in those with p.R227Q (*p* = 0.024). More specifically, of the 88 patients with p.R227Q, 71 had mild or moderate phenotypes, and 58 did not have hypospadias or had a non‐severe form; however, of the 41 patients with p.Q6*, 23 had severe phenotypes, and 28 had severe hypospadias. Similarly, the p.R227Q mutation was more common in patients without hypospadias than in those with (16/17 and 72/113, respectively; *p* = 0.012). We also compared the phenotypes in groups homozygous for different mutations (p.R227Q, p.Q6*, p.R246Q, and p.G203S, Figure 4d–f). The group homozygous for p.R227Q exhibited variable and milder phenotypes than the other groups, in which the phenotype was consistently severe, with scrotal or perineal hypospadias; however, this difference with the p.R227Q group was significant only for the p.Q6* and p.G203S groups (*p* = 0.003–0.045), possibly due to the small sample sizes. Remarkably, when we grouped the patients carrying common mutations (p.Q6*, p.R246Q, and p.G203S) by the presence or absence of p.R227Q, we found that the EMS and urethral meatus score were significantly higher in the group with p.R227Q (*p* = 0–0.011) (Figure 4g–i).

The EMS and urethral meatus score showed great consistency at describing the external genitalia of our patients with 5α‐RD, and PL‐SDS showed roughly the same trend. None of the comparisons showed significant differences in gonad location (*p* = 0.051–0.873).

Phenotypic variability was identified within pairs of siblings carrying the same *SRD5A2* variants (Table 4). The A and B pairs were p.Q6*/p.A228T and p.Q6*/p.R246Q, respectively, and had severe phenotypes with female external genitalia. The C and D pairs shared the same genotype (p.R227Q/p.R246Q) and had hypospadias with micropenis. In contrast, in the E1/E2 pair with p.R227Q/p.G203S and the F1/F2 pair with p.R227Q/p.N193S, one sibling had predominately male genitalia, whereas the other had a severe phenotype and was assigned the female sex at birth.

**Table 4:** Genotypes and phenotypes of six sibling pairs

Sibling pair		Genotype	EMS	Urethral meatus	PL‐SDS
A	1	p.Q6*/p.A228T	3	0	−8.43
	2		3	0	−4.33
B	1	p.Q6*/p.R246Q	3	0	−6.14
	2		3	0	−5.29
C	1	p.R227Q/p.R246Q	5.5	0	−2.8
	2		7	1	−2.67
D	1	p.R227Q/p.R246Q	8	2	−6.71
	2		7	1	−3.95
E	1	p.R227Q/p.G203S	7	1	−4.82
	2		2.5	0	−6.14
F	1	p.R227Q/p.N193S	8	2	−5.57
	2		3	0	−8.94

Abbreviations: EMS, external masculinization score; PL‐SDS, penile length‐standard deviation score; Urethral meatus, urethral meatus score.

## 4 DISCUSSION

We described variable phenotypes, endocrine investigations, and mutational spectra and analyzed genotype‐phenotype correlations in a large highly homogenous Chinese single‐center cohort with 5α‐RD.

Of our 130 patients with confirmed 5α‐RD, 14 had isolated micropenis, an uncommon phenotype also reported by others (Gad et al.., 1997; Maimoun et al., 2011). Residual 5α‐reductase activity and/or DHT production via alternative pathways may explain this phenotype (Domenice, Arnhold, Costa, & Mendonca, 2000; Gad et al., 1997; Maimoun et al., 2011). Isolated micropenis may be more common in China (Cheng et al., 2015) than in Italy (Bertelloni et al., 2016), India (Shabir et al., 2015), Turkey (Abaci et al., 2019), and Germany (Sinnecker et al., 1996).

Although increased T/DHT indicates 5α‐RD, the best cutoff is unclear. A cutoff of 10 is widely used (Maimoun et al., 2011), although a value >8.5 may indicate 5α‐RD in neonates (Walter et al., 2010). Cut‐offs at both 10 and 15 showed great sensitivity in indicating 5α‐RD in our study. However, equivocal and false‐negative results have been reported (Ko et al., 2010). In our cohort with a genetically confirmed diagnosis, the T/DHT value was less than 10 in 4 patients and less than 15 in 10 patients. T/DHT may be normal in patients with mild enzyme deficiency or normal 5α‐reductase type I activity (Maimoun et al., 2011). A recent study demonstrated that extremely low 5α‐ to 5β‐reduced steroid metabolite ratios are pathognomonic for 5α‐RD (Chan et al., 2013), but this methodology is not available in our center. In our study, T/DHT did not differ significantly between the groups with and without hypospadias, and stimulated T/DHT values did not correlate significantly with phenotype. These findings may be related to variations in 5α‐reductase type 2 activity with age.

Over 100 *SRD5A2* variants have been recorded to cause 5α‐RD (Human Gene Mutation Database, www.hgmd.cf.ac.uk, 2019.06.13). Consistent with previous studies, missense and nonsense mutations predominated in our cohort, and exons 1 and 4 were predominantly affected (Abaci et al., 2019; Gui et al., 2019). Homozygous *SRD5A2* mutations are far more common than compound heterozygous mutations in countries other than China (Cheng et al., 2015; Gui et al., 2019; Mendonca et al., 2016), which may be ascribable to the vast population, ethnic diversity, and prohibition of consanguineous marriages in China. Reported *SRD5A2* variants differ across ethnic groups: p.R227Q, p.P212R, p.Q126R and p.G34R have been detected in Asians, Mexicans, Portuguese, and Egyptians, respectively (Samtani, Bajpai, Ghosh, & Saraswathy, 2010). Extensive haplotype analysis has not been performed on these mutations to confirm that they arose on a common genetic background, but their prevalence in geographically or culturally linked countries suggests founder mutation effects (Mendonca et al., 2016). Moreover, three current mutations in our cohort, p.R246Q, p.Q6* and p.G203S, have been identified in previous Asian studies, indicating that these mutations are common in Asian patients (Avendano, Paradisi, Cammarata‐Scalisi, & Callea, 2018).

The type of *SRD5A2* variant may significantly influence phenotypic variability. In our cohort, p.R227Q was present in 88/130 patients, including 15 with homozygosity, and was associated with milder and more variable phenotypes than in the groups without p.R227Q. Furthermore, p.R227Q was more common in patients without hypospadias than in those with hypospadias. The high prevalence of p.R227Q may, therefore, have contributed to the higher proportion of patients with isolated micropenis in our Chinese patients than in other ethnic groups. However, patients homozygous for p.Q6*, p.R246Q and p.G203S showed severe phenotypes. The p.Q6* mutation abolishes virtually all enzyme activity (Zhang et al., 2011). Accordingly, three patients homozygous for p.Q6* exhibited severe phenotypes. The p.R246Q and p.R246W mutations have been shown to impair 5α‐reductase activity as a consequence of reduced NADPH binding (Nie, Zhou, Mao, Lu, & Wu, 2011). Two patients homozygous for p.R246Q and the patient with the p.R246Q/p.R246W genotype had severe hypospadias and EMS ≤3. In vitro, approximately 40% of enzymatic activity is retained with the p.G203S mutation (Zhang et al., 2011). Our five patients homozygous for p.G203S exhibited marked feminization, in accordance with earlier works (Maimoun et al., 2011; Zhang et al., 2011). Interestingly, when these mutations were in compound heterozygosity with p.R227Q, the genotypes were variable and milder, indicating that p.R227Q moderates the functional severity caused by other variants.

The data indicate that patients with mutations leading to complete loss of enzyme activity have a consistently severe phenotype, whereas those who retain some residual enzyme activity have a milder and variable phenotype but are susceptible to other factors. Similar results have been further validated in patients with biallelic nonsense mutations and siblings with the same genotype. In our study, patients with biallelic nonsense mutations consistently exhibited severe phenotypes. Siblings with p.R227Q seemed to have greater phenotypic variability, while siblings without this mutation showed a consistently severe phenotype. Variability in the effects of a given mutation on enzyme activity has been documented (Samtani et al., 2010). Factors other than residual 5α‐reductase 2 activity, such as androgen receptor‐mediated signal transduction, circulating and local T concentrations in utero, and environmental factors, may contribute to phenotypic heterogeneity (Abaci et al., 2019).

Additionally, EMS and the severity of micropenis showed good consistency, indicating that genotypes may be linked to the severity of enzymatic damage and initial penile length. Most patients in our cohort underwent oral testosterone undecanoate treatment to increase PL because DHT gel was not available in China. However, the curative effect was variable: some patients reached a normal PL after 3 months of testosterone therapy, while a few patients did not reach the standard even after 9 months of treatment. The variability may also be correlated with genotype, which we will analyze in subsequent studies.

In conclusion, phenotypic abnormalities in Han Chinese patients with 5α‐RD ranged from isolated micropenis to perineal hypospadias. Increased T/DHT was not consistently present or associated with phenotypic severity. The phenotypic variability may be primarily related to differences in *SRD5A2* variants, with some contribution from other factors. The phenotype is consistently severe when the variant causes a complete loss of enzyme activity but is milder and variable when the enzyme retains some residual activity. The unique prevalence of p.R227Q in the Chinese population suggests a putative founder effect, but haplotype analysis is needed to confirm this. The association of p.R227Q with milder phenotypes may contribute to the higher proportion of patients with isolated micropenis in our Chinese cohort.

## 5 LIMITATIONS

Sometimes 17β‐hydroxysteroid dehydrogenase type 3 (17β‐HSD3) deficiency and *SRD5A2* deficiency are easily confused, and the small number of patients in this study were not screened for 17β‐HSD3 deficiency. However, we excluded the possibility of 17β‐HSD3 deficiency in our patients for the following reasons: (1) in our cohort, most patients presented normal T at baseline or after hCG stimulation, suggesting that 17β‐HSD3 enzyme activity was not absent or limited; and (2) both 17β‐HSD3 deficiency and *SRD5A2* deficiency are autosomal recessive disorders, and the incidence of 17β‐HSD3 deficiency is much lower than that of 5α‐RD. Therefore, the probability of both diseases occurring simultaneously is low.

## CONFLICT OF INTEREST

None of the authors has any conflicts of interest to disclose.

## AUTHORS’ CONTRIBUTIONS

Lijun Fan contributed to collecting the data, performing the statistical analysis, and writing the manuscript. Yanning Song, Beibei Zhang, Lele Li and Di Wu provided the clinical data. Chunxiu Gong and Michel Polak revised the manuscript for important intellectual content.

## Supporting information

Table S1

Table S2

Table S3
