# Supplementary material for: Clinical characteristics and genotype‐phenotype correlations of 130 Chinese children in a high‐homogeneity single‐center cohort with 5α‐reductase 2 deficiency
Source: Mol Genet Genomic Med. 2020 Jul 26;8(10):e1431. doi: 10.1002/mgg3.1431 (PMC7549558; doi:10.1002/mgg3.1431)
Supplement: Supplementary file 1 — Table S1 [file MGG3-8-e1431-s001.docx]

**Supplementary Table 1. Normal age-specific stretched penile length in Chinese males^†^**

| Age | Penile length (cm) |
| --- | --- |
| Newborn | 3.18±0.43 |
| 1-12 months | 3.35±0.35 |
| 1 y | 3.45±0.35 |
| 2 y | 3.54±0.34 |
| 3 y | 3.71±0.33 |
| 4 y | 3.82±0.41 |
| 5 y | 3.96±0.36 |
| 6 y | 4.14±0.43 |
| 7 y | 4.21±0.42 |
| 8 y | 4.23±0.48 |
| 9 y | 4.30±0.49 |
| 10 y | 4.42±0.60 |
| 11 y | 4.48±0.67 |
| 12 y | 5.13±1.07 |
| 13 y | 5.54±1.23 |
| 14 y | 6.03±1.40 |
| 15 y | 6.90±1.21 |
| 16 y | 7.12±1.22 |
| 17 y | 7.26±1.16 |
| 18 y | 7.33±1.06 |
| Adult | 8.17±0.97 |

^†^(Fu & Li, 2010).
