# Supplementary material for: Clinical characteristics and genotype‐phenotype correlations of 130 Chinese children in a high‐homogeneity single‐center cohort with 5α‐reductase 2 deficiency
Source: Mol Genet Genomic Med. 2020 Jul 26;8(10):e1431. doi: 10.1002/mgg3.1431 (PMC7549558; doi:10.1002/mgg3.1431)
Supplement: Supplementary file 2 — Table S2 [file MGG3-8-e1431-s002.docx]

**Supplementary Table 2. *SRD5A2* gene variants identified in 130 Chinese children with 5α-RD**

| **No.** | **Variant** | **E or I** | **Type** | **ACMG** | **Hom** | **Het** | **No. of alleles** |
| --- | --- | --- | --- | --- | --- | --- | --- |
| 1 | **c.680G>A (p.R227Q)** | E4 | M | P | 15 | 73 | 103 |
| 2 | **c.16C>T (p.Q6*)** | E1 | N | P | 3 | 38 | 44 |
| 3 | **c.737G>A (p.R246Q)** | E5 | M | P | 2 | 31 | 35 |
| 4 | **c.607G>A (p.G203S)** | E4 | M | P | 5 | 17 | 27 |
| 5 | c.578A>G (p.N193S) | E4 | M | LP | 0 | 7 | 7 |
| 6 | c.100G>C (p.G34R) | E1 | M | P | 0 | 4 | 4 |
| 7 | c.683C>T (p.A228V) | E4 | M | LP | 0 | 4 | 4 |
| 8 | c.59T>C (p.L20P) | E1 | M | LP | 0 | 3 | 3 |
| 9 | c.211C>T (p.Q71*) | E1 | N | P | 0 | 3 | 3 |
| 10 | c.656delT (p.F219Sfs*60) | E4 | F | P | 0 | 3 | 3 |
| 11 | c.105G>C (p.K35N) | E1 | M | LP | 0 | 2 | 2 |
| 12^†^ | c.268C>T (p.H90Y) | E1 | M | LP | 0 | 2 | 2 |
| 13 | c.307C>T (p.S103*) | E2 | N | P | 1 | 0 | 2 |
| 14 | c.663delT (p.C222Pfs*11) | E4 | F | P | 0 | 2 | 2 |
| 15 | c.682G>A (p.A228T) | E4 | M | LP | 0 | 2 | 2 |
| 16 | c.736C>T (p.R246W) | E5 | M | P | 0 | 2 | 2 |
| 17 | c.89dupC (p.S31fs*105) | E1 | F | P | 0 | 1 | 1 |
| 18^†^ | c.154C>G (p.A52P) | E1 | M | LP | 0 | 1 | 1 |
| 19^†^ | c.171G>C (p.E57D) | E1 | M | LP | 0 | 1 | 1 |
| 20^†^ | c.173A>C (p.L58R) | E1 | M | LP | 0 | 1 | 1 |
| 21 | c.196G>A (p.G66R) | E1 | M | LP | 0 | 1 | 1 |
| 22^†^ | c.205G>C (p.A69P) | E1 | M | LP | 0 | 1 | 1 |
| 23^†^ | c.247C>A (p.L83I) | E1 | M | LP | 0 | 1 | 1 |
| 24^†^ | c.269A>G (p.H90R) | E1 | M | LP | 0 | 1 | 1 |
| 25 | c.371T>A (p.V124D) | E2 | M | LP | 0 | 1 | 1 |
| 26^†^ | c.374T>G (p.L125R) | E2 | M | LP | 0 | 1 | 1 |
| 27 | c.419G>A (p.W140*) | E2 | N | P | 0 | 1 | 1 |
| 28 | c.433C>T (p.R145W) | E2 | M | LP | 0 | 1 | 1 |
| 29^†^ | c.650C>A (p.A217E) | E4 | M | LP | 0 | 1 | 1 |
| 30^†^ | c.705C>A (p.Y235*) | E | N | P | 0 | 1 | 1 |
| 31 | c.282-2A>G | I1 | S | P | 0 | 1 | 1 |
| Total | |  |  |  |  |  | 260 |

**Abbreviations:** E, exon; I, intron; hom, homozygous condition; het, compound heterozygous condition; No. of alleles, total number of alleles; M, missense mutation; N, nonsense mutation; F, frameshift variant; S, splice site variant; ACMG, American College of Medical Genetics and Genomics and the Association for Molecular Pathology guidelines; P, pathogenic; LP, likely pathogenic. ^†^novel variant.
