# Supplementary material for: Clinical characteristics and genotype‐phenotype correlations of 130 Chinese children in a high‐homogeneity single‐center cohort with 5α‐reductase 2 deficiency
Source: Mol Genet Genomic Med. 2020 Jul 26;8(10):e1431. doi: 10.1002/mgg3.1431 (PMC7549558; doi:10.1002/mgg3.1431)
Supplement: Supplementary file 3 — Table S3 [file MGG3-8-e1431-s003.docx]

**Supplementary Table 3. Molecular characteristics and in silico analysis of the identified novel variants in the *SRD5A2* gene**

| **No.** | **Variant** | **ACMG** | **Polyphen2 result** |
| --- | --- | --- | --- |
| 1 | c.154C>G (p.A52P) | LP (PM2 PM3 PP3 PP4) | Possibly damaging |
| 2 | c.171G>C (p.E57D) | LP (PM2 PM3 PP3 PP4) | Possibly damaging |
| 3 | c.173A>C (p.L58R) | LP (PM2 PM3 PP3 PP4) | Possibly damaging |
| 4 | c.205G>C (p.A69P) | LP (PM2 PM3 PP3 PP4) | Possibly damaging |
| 5 | c.247C>A (p.L83I) | LP (PM2 PM3 PP3 PP4) | Possibly damaging |
| 6 | c.268C>T (p.H90Y) | LP (PM2 PM3 PP3 PP4) | Probably damaging |
| 7 | c.269A>G (p.H90R) | LP (PM2 PM3 PP3 PP4) | Probably damaging |
| 8 | c.374T>G (p.L125R) | LP (PM2 PM3 PP3 PP4) | Probably damaging |
| 9 | c.650C>A (p.A217E) | LP (PM2 PM3 PP3 PP4) | Probably damaging |
| 10 | c.705C>A (p.Y235*) | P (PVS1 PM3 PP3 PP4) | - |

**Note:** ACMG, American College of Medical Genetics and Genomics and the Association for Molecular Pathology (ACMG/AMP) guidelines; LP, likely pathogenic; P, pathogenic.
